# Factors and Perceptions Associated with Post-Pandemic Food Sourcing and Dietary Patterns among Urban Corner Store Customers in Baltimore, Maryland

**DOI:** 10.3390/nu16142196

**Published:** 2024-07-10

**Authors:** Emma C. Lewis, Yutong Xie, Samantha M. Sundermeir, Lisa Poirier, Stacey Williamson, Sarah Lee, Xinyue Pei, Jennifer Stephenson, Antonio J. Trujillo, Takeru Igusa, Joel Gittelsohn

**Affiliations:** 1 Department of International Health, Johns Hopkins Bloomberg School of Public Health, 615 N. Wolfe Street, Baltimore, MD 21205, USA; srex2@jh.edu (S.M.S.); lpoirie4@jh.edu (L.P.); swill363@jh.edu (S.W.); slee464@jh.edu (S.L.); peixwork@gmail.com (X.P.); jstephenson@nas.edu (J.S.); atrujil1@jhu.edu (A.J.T.); jgittel1@jhu.edu (J.G.); 2 Department of Epidemiology, Johns Hopkins Bloomberg School of Public Health, 615 N. Wolfe Street, Baltimore, MD 21205, USA; yxie62@jh.edu; 3 Department of Civil and Systems Engineering, Johns Hopkins Whiting School of Engineering, 3400 N. Charles Street, Baltimore, MD 21218, USA; tigusa1@jhu.edu

**Keywords:** urban, Baltimore, food sourcing, food environment, dietary quality, COVID-19, corner stores

## Abstract

Objective. Diet-related disease is rising, disproportionately affecting minority communities in which small food retail stores swamp supermarkets. Barriers to healthy food access were exacerbated by the pandemic. We examined the following: (1) individual- and household-level factors in a sample of Baltimore community members who regularly shop at corner stores and (2) how these factors are associated with indicators of dietary quality. Design. Cross-sectional data were collected using an online survey to capture sociodemographics, anthropometrics, and food sourcing, spending, and consumption patterns. Concurrent quantitative and qualitative analyses were conducted in Stata 18 and ATLAS.ti. Setting. This study was set in Baltimore, Maryland, USA. Participants. The participants included adults (*n* = 127) living or working in Baltimore who identified as regular customers of their neighborhood corner store. Results. The respondents were majority Black and low-income, with a high prevalence of food insecurity (62.2%) and overweight/obesity (66.9%). Most (82.76%) shopped in their neighborhood corner store weekly. One-third (33.4%) of beverage calories were attributed to sugar-sweetened beverages, and few met the recommended servings for fruits and vegetables or fiber (27.2% and 10.4%, respectively). Being Black and not owning a home were associated with lower beverage and fiber intake, and not owning a home was also associated with lower fruit and vegetable intake. Food insecurity was associated with higher beverage intake, while WIC enrollment was associated with higher fruit and vegetable and fiber intakes. Open-ended responses contextualized post-pandemic food sourcing and consumption in this setting. Conclusions. This paper helps characterize the consumers of a complex urban food system. The findings will inform future strategies for consumer-engaged improvement of local food environments.

## 1. Introduction

Nearly 60% of United States adults are currently living with at least one chronic disease (e.g., obesity, cancer, diabetes), and this rate is projected to increase over the next three decades [1,2]. Diet contributes to the development of many chronic diseases, and the presence of at least one increases the risk for multimorbidity [3]. The chronic disease burden disproportionately affects minority populations because of the complexity of historical, social, economic, and environmental mechanisms [4]. Members of these populations are more likely to reside in under-resourced communities with high access to energy-dense foods and sugar-sweetened beverages, which increase the risk for chronic disease, and low access to fruits and vegetables, which are protective for chronic disease [5]. These communities, often referred to as “food swamps”, tend to be characterized by an abundance of small independently owned food retail stores (“corner stores”) and prepared food sources (“carryouts”), and a lack of nearby supermarkets. Strong linkages have been made among inequitable food environments, food access (e.g., availability and affordability), and dietary quality as predictors of disease [5]. In recent years, the coronavirus disease of 2019 (COVID-19) pandemic further exacerbated existing inequities, as resources were stretched thin for many American families [6].

Many interventions, programs, and policies have sought to improve food access under usual and emergency (e.g., pandemic) conditions, although most have focused extensively on one level of the food system, failing to address root causes of diet-related health disparities. There has been a recent call to action for research to develop and implement strategies with a broader focus on the food system as a whole across local, state, and federal levels [7]. Several examples of successful multi-level multi-component intervention trials further support this requisition, some of which demonstrated particular promise in Baltimore, Maryland, prior to COVID-19 [8,9,10,11].

In 2020, the Baltimore Urban food Distribution (BUD) study received funding to improve the food distribution system for healthy, affordable foods and beverages among Baltimore corner stores and local farmers, producers, wholesalers, and distributors [12]. The BUD intervention leverages a digital tool—a timely innovation given the rise in the use of digital health technology during COVID-19—referred to as the BUD mobile application (“BUD app”). The app is designed to allow for a streamlined exchange of hyperlocal items between retailers and suppliers [13,14]. While the intervention primarily focuses on engaging retailers and suppliers in the supply-side proliferation of healthy foods, the app can be expanded to involve consumers in the demand-side feedback loop for reinforced stocking and purchasing of these foods in their community environments.

As of January 2024, the Maryland Food Bank estimates that grocery prices have increased by nearly 20% since January 2020 [15]. To that end, acquiring fresh, nutritious foods has become increasingly challenging for Baltimore residents, especially those navigating neighborhoods where generational disparities in food access and health persist. An updated understanding of who the consumers in this food system are, and how their food sourcing patterns and perceptions play a role in their diets, could inform future intervention strategies including the integration of consumer input in the BUD app and parameterization of an agent-based model to simulate chronic disease risk. Therefore, the present paper sought to explore the following research questions:

1. What are the individual- and household-level factors characterizing a sample of Baltimore community members who regularly shop in their neighborhood corner stores?
2. How are these individual- and household-level factors associated with indicators of dietary quality (i.e., sugar-sweetened beverage consumption, fruit and vegetable intake, and dietary fiber intake)?

## 2. Methods

### 2.1. Study Setting

Baltimore, Maryland, is an example of an inequitable urban food system that contributes to differential food access among residents. In 2018, it was estimated that nearly one-quarter of residents (23.5%) lived in areas deemed to be “Healthy Food Priority Areas”—more commonly recognized as food deserts or swamps—where healthy food availability, transportation, annual household income, and the presence of supermarkets are low [16]. Racial/ethnic minority residents are more likely to live in these areas and disproportionately lack adequate access to food compared to their White counterparts. While there were 47 supermarkets and 6 public markets in Baltimore prior to the pandemic, small independently owned corner stores and convenience stores are much more ubiquitous, amounting to over 700 locations (525 corner stores and 183 convenience stores) citywide [16]. Given the absence of supermarkets in many neighborhoods, residents often frequent these smaller stores for food purchases.

### 2.2. Sampling

Respondents (*n* = 127) were adults who (1) live or work within a 0.5-mile radius of a corner store; (2) purchase food items at least once per week from a corner store; (3) are 21–75 years old; (4) plan to stay in Baltimore for at least the next 12 months; and (5) are not pregnant. The age range for eligible respondents was selected to capture adults who would be most likely to have high individual purchasing power at small stores. Recruitment was carried out via in-person flyers, social media, and word-of-mouth (e.g., snowball sampling), including follow-up emails sent to respondents encouraging the referral of local friends, family, and colleagues. A description of the original study protocol can be found in Gittelsohn and Colleagues (2022), although the data collection strategies described here reflect a later shift from in-person to online because of pandemic-related restrictions [12,17].

### 2.3. Materials and Measures

Data were collected between March and September 2023 using a modified online version of an Adult Impact Questionnaire (AIQ) fielded in previous studies in Baltimore [12,18]. The instrument consisted of 84 closed- and open-ended questions including dietary intake screeners selected to capture foods belonging to the categories promoted as part of the larger BUD trial including (1) beverages (2) fruits and vegetables, and (3) whole grains/fiber. The AIQ was administered using Qualtrics and made available for mobile and web through a secure Johns Hopkins University server. On average, respondents spent 50.76 min completing the survey.

#### 2.3.1. Quantitative Measures

Individual factors. Sociodemographic information. Individual-level sociodemographic information was gathered including home address (to capture neighborhood and proximity to BUD-participating corner stores), age in years, gender, race/ethnicity, marital status, level of highest education, and employment status. Food sourcing. Patterns and behaviors related to food sourcing were collected through questions pertaining to how many times certain foods and beverages were bought over the last 30 days and from which types of food sources they were purchased. Psychosocial factors. Psychosocial questions pertaining to healthy eating were used to assess behavioral intentions, outcome expectancies, and self-efficacy. For example, selected items were used as prompts for future purchasing intentions, such as “The next time you purchase bread, which would you choose?” with pre-selected options (e.g., white bread, potato bread, 100% whole wheat bread). Anthropometry. Respondents were asked questions regarding their blood pressure, weight, and height. Body Mass Index (BMI) was calculated using self-reported height in inches and self-reported weight in pounds as measured by a medical professional or at home. If the respondent provided their weight as measured by a medical professional, that number was utilized. Using the formula: weight (Ib)/[height (in)]^2^ × 703, BMI was recorded in Excel Version 16.85 and categorized in Stata 18 as either underweight (BMI less than 18.5), normal (BMI between 18.5 and 24.9), overweight (BMI between 25 and 29.9), obese (BMI between 30 and 39.9), or severely obese (BMI of 40 or over) [19].

Household factors. Household-level information was assessed through questions pertaining to socioeconomic status (e.g., number of adults and children living in the household, annual household income, housing arrangement, and household participation in food assistance programs), as well as questions on food purchasing and food preparation. Household food security status was obtained using the U.S. Household Food Security Survey Module: Six-Item Short Form [20]. Responses to the six questions were scored to produce a sum total raw score ranging from 0 to 6. A raw score of 0–1 was considered “high or marginal food security”, a raw score of 2–4 was considered “low food security”, and a raw score of 5–6 was considered “very low food security”. In other words, respondents with a score from 2 to 6 were generally food insecure.

Dietary quality indicators. Beverage intake. Beverage intake was measured using the Beverage Intake Questionnaire (BEVQ-15) [21,22]. The questionnaire asks respondents to indicate approximately (1) how often and (2) how much each time, they drank fifteen researcher-selected beverages, including sugar-sweetened beverages, in the past month. Responses are scored into average daily fluid ounces, average daily calories, and average daily grams consumed for each beverage, and then these scores are summed to find the total average daily fluid ounces, total average daily calories, and total average daily grams for all beverages. Fruit and vegetable intake. Fruit and vegetable intake was measured using the Block Fruit/Vegetable/Fiber (FVF) Screener [23,24]. The screener includes 7 questions about fruit and vegetable intake and 3 questions about foods high in fiber. Responses are used to rank individuals with regard to their usual intake, producing scores ranging from 0 to 35 for fruits and vegetables (with a score of less than 11 being considered “low”) and from 0 to 50 for fruits, vegetables, and beans, as well as a point estimate of total daily fruit and vegetable servings. Fiber intake. Fiber intake from fruits and vegetables, beans, and whole grains was also measured using the Block FVF Screener, which, in addition to producing a score associated with fruit and vegetable intake, produces point estimates for various daily nutrient intakes including vitamin C (mg), magnesium (mg), potassium (mg), and dietary fiber (mg).

#### 2.3.2. Qualitative Measures

COVID-19 impact. Respondents were asked two qualitative open-ended questions as follows: (1) “In 1–2 sentences, can you tell us about how the COVID-19 pandemic has impacted how and where you food shop? What foods do you shop for? (Please respond with at least 10 words.)” and (2) “In 1–2 sentences, do you think the COVID-19 pandemic has changed how people in your community shop for food? What about how stores in your community sell food? (Please respond with at least 10 words)”. The questions were designed to prime respondents for the thoughtfulness of both facilitators and barriers to food sourcing as they completed the AIQ, as well as to supplement the quantitative data with the shared lived experiences of respondents.

### 2.4. Data Checking

Given the nature of online data collection, extensive data checking was required because of the infiltration of bots and scammers. We developed a comprehensive two-part, three-reviewer protocol based on recommendations from Griffin and Colleagues (2022) who reported experiencing similar challenges [25].

### 2.5. Data Analysis

A concurrent nested triangulation approach to data analysis was used, whereby the quantitative and qualitative data were collected at the same time during the administration of the AIQ but analyzed separately with more weight given to the quantitative analyses [26]. Concurrent triangulation designs allow researchers to define relationships more accurately among variables of interest and to recontextualize theory in other populations and settings [27,28]. Descriptive and statistical quantitative analyses were conducted using Microsoft Excel for Mac (version 16.81) [29] and Stata (version 18) [30]. Thematic qualitative analysis was conducted using ATLAS.ti Web (version 24) [31].

Descriptive analysis. Respondent characteristics were analyzed using descriptive statistics in Stata. Variables were created to collapse or dichotomize measures of interest, if appropriate. All dietary data were scored based on guidelines provided by the developers of each screener tool. Independent samples *t*-tests and Wilcoxon–Mann–Whitney tests were used to compare the means of dependent dietary intake variables among dichotomous independent factors of interest. Chi-square tests and one-way analysis of variance (ANOVA) were used to examine associations among categorical sociodemographic variables.

Statistical analysis. A linear regression model-building approach was used to analyze the associations between categorical measures of interest, or factors, and continuous dietary intake outcomes. Three models were constructed beginning with sociodemographic factors, then adding additional individual-level factors, and finally, adding household-level factors. This was repeated three times for each of the three dietary outcomes of interest (daily beverage intake, daily servings of fruits and vegetables, and daily dietary fiber intake), which were each regressed on different independent variables. Assumptions of normality were investigated, as well as model specification and backward stepwise selection, and independent *t*-tests were used to select the final model of best fit for each outcome of interest, taking multicollinearity into account. The variance inflation factor (VIF) was employed to check for multicollinearity within each regression model with a tolerance (defined as 1/VIF) level set to 0.1. For all analyses, statistical significance was defined by a *p*-value of <0.05.

Thematic analysis. An inductive approach to coding was employed by a primary (Y.X.) and secondary (E.C.L.) coder. The coding process followed a similar process to that of Grounded Theory, such that codes were derived directly from the question responses first and then condensed into a shorter list of codes applied to each response [32]. Codes were further examined for those with few quotations and, in some instances, were combined with other codes. The codes were categorized into two overarching groups—“Individual-Level Changes” and “Store/Community-Level Changes”—based on the premises of the two open-ended response questions, and the resulting key themes were then analyzed in ATLAS.ti.

## 3. Results

### 3.1. Description of the Study Sample

Individual-level factors. The respondents (*n* = 127) ranged in age from 21 to 67 years old (mean (M) = 38.95 years, standard deviation (SD) = 11.27). The majority reported being female (65.4%) and Black or African American (51.2%). A little less than half (46.5%) were married, about one-quarter (23.6%) held a bachelor’s degree, and around two-thirds (66.9%) were currently employed. Two-thirds (66.9%) were overweight or obese based on calculated BMI scores. Overall, 82.76% reported shopping at a neighborhood corner store at least once per week—while 16.55% reported daily corner store shopping, and only 66.90% reported shopping in a supermarket at least once per week. When asked about food purchasing intentions for staple items such as milk, bread, and rice, only one-quarter (25.20%) said they would choose 1% low-fat milk over higher-fat options (e.g., 2% milk, whole milk), one-third (34.65%) said they would choose 100% whole wheat bread over potato or white bread, and a little less than one-quarter (21.26%) said they would choose brown rice over white or yellow rice at their next purchase. About forty-five percent (44.83%) indicated intention to purchase the healthiest option for all three items.

Household-level factors. Most (63.8%) rented their household residence and had a mean annual household income of less than USD 40,000—and more than eighty percent (81.1%) reported an annual household income that was below the median annual household income level for Baltimore (USD 54,735) [33]. Regarding food spending, respondents spent, on average, USD 710.31 total on food in the last 30 days, of which an average of USD 238.05 came from food assistance, to feed a mean number of three people per household. Seventy-two percent (72.44%) reported having at least one (M = 1.21, SD = 0.98) child under 18 years of age living in the household. More than half (57.48%) reported that someone in their household had participated in the Special Supplemental Nutrition Program for Women, Infants, and Children (WIC) within the past year, followed by 39.37% in free or reduced-cost school breakfast, 34.65% in the Supplemental Nutrition Assistance Program (SNAP), and 14.17% in free or reduced-cost school lunch. Almost two-thirds (62.2%) shared that their household had experienced food insecurity (low or very low food security) in the last year.

Relationships among factors. Being Black was significantly associated with food insecurity status (F(2, 123) = 3.87, *p* = 0.023). A Tukey post hoc test revealed that the likelihood of being Black was significantly associated with low food security (0 = 0.043) or very low food security (*p* = 0.064) compared with high or marginal food security. Being Black was also significantly associated with BMI status (F(5, 120) = 3.83, *p* = 0.003), especially for those in obesity classes II (*p* = 0.003) and III (*p* = 0.046) compared with those considered normal weight, and for those in obesity class II compared with those considered overweight (*p* = 0.039).

Not surprisingly, the highest level of education obtained was significantly associated with employment status (F(2, 124) = 20.02, *p* = 0.0001) and annual household income (F(2, 124) = 25.61, *p* = 0.0001). WIC enrollment was significantly associated with food security status (F(2, 124) = 4.29, *p* = 0.016), and this was seen particularly for those who had very low food security compared with high or marginal food security (*p* = 0.012). Participating in the SNAP program, however, was not associated with food security in this sample.

Regarding dietary intake, the average daily caloric intake from beverages was 1372.68 calories (SD = 1240.004), 458.37 calories (SD = 423.47) of which were attributed to consumption of sugar-sweetened beverages (SSBs) on average. Estimated daily servings of fruits and vegetables was 3.78, on average (SD = 2.06), which is only 75.6% of the recommended daily serving size of 5 [34]. In fact, only 27.2% of the sample had a predicted daily serving size of 5 or more servings. Predicted daily intake of fiber was 15.13 g on average (SD = 7.09). This is similar to the national average for adults, which falls around 15 g of fiber per day, although the United States Department of Agriculture (USDA) recommends that adults up to age 50 consume 25 g for women and 38 g for men, per day, and those above 50 consume 21 g for women and 30 g for men [35]. In our sample, across all adults, only 10.4% were estimated to consume 25 g or more of fiber daily.

A summary of respondent characteristics across food and nutrient intakes can be found in Table 1.

### 3.2. Associations with Beverage, Fruit and Vegetable, and Fiber Intake

Daily calories from beverages. In the final model, being Black (β = −456.74, CI: −891.73, −21.74, *p* = 0.040) and not owning a home (β = −349.67, CI: −695.64, −3.71, *p* = 0.048) were significantly associated with a lower daily intake of calories from beverages. Being at greater risk for food insecurity (β = 592.87, CI: 99.43, 1086.32, *p* = 0.019) and having at least one child under 18 years of age in the home (β = 541.48, CI: 66.87, 1016.08, *p* = 0.026) were significantly associated with higher daily intake of calories from beverages. WIC enrollment, annual household income, future intention for purchasing rice, and high blood pressure were not significantly associated with daily beverage intake.

Daily servings of fruits and vegetables. In the final model, not owning a home (β = −0.58, CI: −1.07, −0.09, *p* = 0.017) was significantly associated with lower daily servings of fruits and vegetables. Having a higher level of education (β = 0.51, CI: 0.12, 0.91, *p* = 0.012) and WIC enrollment (β = 1.07, CI: 0.37, 1.78, *p* = 0.003) were significantly associated with higher daily servings of fruits and vegetables. Being Black, overweight/obese, male, household size, and spending more food money on fruits and vegetables were surprisingly not significantly associated with daily fruit and vegetable intake.

Daily dietary fiber intake. In the final model, being Black (β = −2.40, CI: −4.39, −0.40, *p* = 0.019), being older (β = −2.40, CI: −4.38, −0.42, *p* = 0.018), and not owning a home (β = −1.77, CI: −3.19, −0.35, *p* = 0.015) were significantly associated with a lower daily intake of dietary fiber. Being male (β = 6.12, CI: 4.02, 8.22, *p* = 0.0001) and WIC enrollment (β = 3.22, CI: 1.16, 5.28, *p* = 0.003) were significantly associated with a higher daily intake of dietary fiber. Being overweight/obese, having a higher level of education, and spending more food money on fruits and vegetables were not significantly associated with daily fiber intake.

All factors included in each of the three final linear models can be found in Table 2.

### 3.3. Perceptions of COVID-19 Impacts on Food Sourcing and Consumption

Twenty-two key themes were identified as representing community member perspectives of COVID-19 impacts on food sourcing and consumption. Examples of each theme can be found in Table 3 and are included in a discussion of the study findings in the following section.

## 4. Discussion

This paper examines individual- and household-level factors and community member perceptions associated with dietary quality (beverage, fruit and vegetable, and dietary fiber intake) in the wake of pandemic-related disruptions to small urban food retailers. We found that one-third of daily average beverage consumption was attributed to sugar-sweetened beverages, and few adults met the recommended daily intakes for fruits and vegetables and fiber. Linear regression models revealed significant associations among factors such as being Black, home ownership, WIC program enrollment, and indicators of dietary quality. Community member perceptions codified across 22 themes pointed to several implications for food sourcing and consumption patterns given recent shifts in the food system.

Most (82.8%) respondents reported shopping in their neighborhood corner store at least once per week, citing proximity and convenience as major factors in their selection of food retail sources. In regard to diet, one-third (33.4%) of daily average beverage calories consumed were attributed to sugar-sweetened beverages, less than 30 percent (27.2%) met the recommended amount of daily fruit and vegetable servings, and only 10 percent (10.4%) consumed the minimum recommended amount of dietary fiber per day. Mentions of COVID-19’s impact on the selection of types and quality of foods pertained to a lack of fresh items and the need to shift towards more processed, shelf-stable, and canned goods. Importantly, processed and ultra-processed foods are commonly higher in added sugars and lower in fiber, protein, vitamins, and other nutrients compared with whole, nutritious foods [36]. Ultra-processed foods in particular may contribute towards empty calories, displacing more nutrient-dense foods and leading to a calorically dense yet undernourished diet [36].

Interestingly, among our sample, being Black and not owning a home were associated with lower daily beverage and fiber intake, and not owning a home was also associated with lower fruit and vegetable intake. Homeownership is an important indicator of wealth accumulation over time and may affect health through various mechanisms, especially for low-income households [37]. Individuals with greater wealth have been shown to experience lower mortality, higher life expectancy, and decreased risk of chronic diseases including obesity, hypertension, and asthma [38]. A 2018 report highlighted several key findings linking greater wealth with better health, including that wealth is associated with healthier living conditions and access to health care and is protective against chronic stress—which is known to negatively impact diet and mental and physical health [39]. Moreover, home ownership may suggest greater housing stability, which could be protective for dietary quality. In one study, housing instability was associated with lower vegetable consumption and lower overall dietary quality among an urban adult population [40]. However, the existing literature on specific mechanisms underlying home ownership and its impact on diet in the United States is mixed, with most studies focused on income and broader health outcomes. Given that not owning a home was associated with a lower intake of all three dietary groups assessed in our sample, further investigation of potential mechanisms is warranted, and local, state, and federal homeownership programs should be considered as part of future systems interventions aimed at improving healthy food access and diet.

Not surprisingly, food insecurity was associated with higher daily caloric intake from beverages—one-third (33.39%) of which came from sugar-sweetened beverages, on average—and WIC enrollment was associated with higher daily fruit and vegetable and fiber consumption. Previous studies in the literature have identified food insecurity as a potential driver of sugar-sweetened beverage consumption across child, adolescent, and adult populations [41,42,43]. This relationship has been observed both inside and outside of the home—a 2019 study found that the odds of purchasing a sugar-sweetened beverage at a small or top chain restaurant were higher in households with marginal and low food security, and the odds of purchasing a low-calorie beverage were lower in households with very low food security [44]. Previous studies also support the finding that WIC program enrollment is associated with increased consumption of fruits and vegetables and dietary fiber. Most recently, the increased WIC cash value benefit (CVB) was found to be associated with a greater amount and diversity of redeemed fruits and vegetables among participants of the program [45]. In addition, according to a 2021 report, WIC participants buy and eat more fruits and vegetables, as well as whole grains and low-fat dairy products [46]. However, national WIC enrollment rates remain low, with more than half of WIC-eligible recipients not enrolled in the program as of 2021 [47]. In Maryland, the enrollment rate for all eligible recipients dropped from 59.8% to 55.4% from 2020 to 2021 [47,48]. Therefore, future interventions in this setting should consider ways in which to increase WIC enrollment and retention for eligible households. WIC benefits redemption could be explored in small independently owned corner stores and other small-box stores (e.g., dollar stores), as well, in order to improve the selection of healthy options given the program’s minimum stocking requirements.

When asked about food sourcing and consumption in their communities, many respondents shared that their nearby stores lacked variety in the healthy items available, and stores that did offer them were often located further away or had higher prices. This seemed to be exacerbated by the pandemic, with some respondents mentioning shifts in their food shopping behaviors, such as sourcing shelf-stable canned fruits and vegetables and utilizing online food services that offer coupons or discounts. Respondents also expressed concerns regarding the quality of foods stocked in corner stores. Several sentiments placed the blame on corner stores, although others acknowledged the influence of broader food system challenges due to the pandemic. A desire for convenience, accessibility, and close proximity to stores was brought up frequently by community members, sometimes overriding the desire for healthy options. Interestingly, a few respondents mentioned having relationships with small store owners, and some highlighted the importance of community togetherness and supporting local businesses. Support from local food assistance programs, such as food pantries, tended to be associated with feelings of community togetherness, especially for those hit by harder times during the pandemic.

Coupled with our findings pertaining to dietary quality and associated characteristics, these respondent perspectives provided a deeper understanding of the complex food shopping experiences of those navigating a disrupted urban food environment. For example, it is possible that the lower overall fruit and vegetable intake could be partly explained by the challenges related to availability, variety, quality, and price discussed by respondents. In addition, lower intake of dietary fiber could be due in part to increased purchasing of canned and processed foods, which are more likely to be lower in fiber and other nutrients than fresh or frozen foods. Mention of relationships with small, neighborhood store owners could be leveraged in the BUD study—in fact, a consumer module (called “BUDConnect”) of the BUD app is currently being developed and tested to provide a digital platform for relationship-building between store owners and their regular customers. Therefore, these findings will help ensure community members’ needs are adequately met through BUDConnect, such as being able to request the stocking of desired healthy items and rate and review the quality and pricing across various neighborhood stores.

The present study had several challenges worth noting. Online recruitment required the need to develop and implement an added step to our data-checking protocol given the nature of online bots. In addition, the data collected were cross-sectional and included self-reported anthropometric measures, which were used to calculate BMI and may have introduced opportunities for bias. However, most recent studies agree that self-reported height and weight are sufficient for use in research [49]. Finally, daily caloric intake from beverages was high among one-fifth (18.9%) of the sample relative to the average estimated total daily caloric intake in the United States. This could be due in part to the self-report nature of the BEVQ-15 instrument, although we did not capture total daily caloric intake in our sample and were therefore limited in our ability to contextualize unexpectedly high average intakes of dietary components with regard to respondents’ overall diet. Future research should take this into consideration, in addition to our sample, which was limited in size.

Given recent calls to action for research to develop and implement informed, broad-scale programs and policies for improved nutrition and health [7], our next steps will include parameterizing an agent-based simulation model. Simulation models have been used to represent flows and accumulated stocks of healthy foods using modern representations of urban food systems, including Baltimore [50]. In this case, the planned model will utilize our AIQ data and other existing data sources to represent the impact of food environment programs and policies on dietary behaviors, obesity, and cancer risk. Once calibrated and tested, this tool can provide policymakers with guidance in the selection of evidence-based strategies for improving outcomes and reducing the burden of disease. To our knowledge, a model of this nature does not currently exist but could play a crucial role in future preparedness and response to both usual and emergency disruptions to the food system.

## 5. Conclusions

The pandemic exacerbated existing issues of food access and food security for vulnerable communities nationwide, introducing nuanced challenges for sourcing and consuming healthy foods and beverages. Diet-related chronic disease risk continues to rise, underscoring a critical need for effective and sustainable interventions, programs, and policies that target the consumer, retailer, and supplier levels of the food system simultaneously. Consumers play a particularly important role in the supply–demand feedback loop for stocking and purchasing in small food retail stores, and the factors implicated in this complex system require better understanding. The findings presented here fill a critical gap in the literature and help inform intervention strategies such as the design and testing of an app to engage consumers in the demand-side proliferation of healthy food access and a simulation model for policymakers to assess potential risks and benefits of future programs and policies.

## Figures and Tables

**Table 1:** Daily food and nutrient intakes by individual- and household-level characteristics.

		Daily Food and Nutrient Intake		
		Beverages, Kcal	Fruits and Vegetables, Servings	Total Fiber, Grams
Characteristics	*n* = 127	Mean (SD)	Mean (SD)	Mean (SD)
Individual level				
Gender				
Male	42	1514.36 (1162.00)	4.29 (2.15) *	19.65 (6.19) ***
Female ^∇^	83	1307.25 (1290.62)	3.52 (1.98)	12.84 (6.42)
Non-Binary	2	1113.04 (676.92)	-	-
Age (years)				
21–34 ^∇^	50	1319.68 (1118.45)	3.98 (2.22)	17.34 (6.62)
35–44	45	1611.52 (1424.05)	3.74 (1.75)	15.66 (6.50)
45–54	13	878.85 (765.54)	2.85 (2.37)	10.49 (7.95) **
55–64	17	1402.08 (1318.90)	3.88 (2.18)	11.22 (6.94) **
65–75	2	283.89 (294.32)	5.01 (1.18)	13.73 (1.05)
Race/ethnicity				
Black or African American ^∇^	65	1101.86 (1057.52)	3.20 (1.96)	12.71 (6.97)
White	49	1649.27 (1322.39) *	4.68 (2.06) ***	18.41 (6.81) ***
Other	13	1724.13 (1601.13)	3.40 (1.47)	14.95 (3.06)
Marital status				
Married ^∇^	59	1766.02 (1275.38)	4.69 (1.93)	19.24 (5.97)
Never Married	49	953.20 (1009.41) ***	2.79 (1.85) ***	11.14 (6.02) ***
Divorced	6	1746.00 (2257.14)	3.01 (1.31) *	12.00 (5.00) **
Other	13	996.33 (618.23) *	3.57 (1.99)	12.34 (6.31) ***
Education				
Less than 12th grade ^∇^	10	1899.49 (2010.92)	2.88 (1.55)	13.25 (7.25)
High School or GED	32	1112.63 (997.78)	2.84 (2.01)	11.59 (6.81)
Less than 2 years of college or vocational school	31	971.83 (639.77) *	3.65 (1.68)	14.02 (5.44)
Associate or bachelor’s degree	42	1687.87 (1426.35)	4.65 (2.20) *	18.58 (7.17) *
Graduate School	10	1649.74 (1300.68)	4.36 (1.87)	17.48 (6.33)
Other	2	1108.17 (750.20)	4.96 (0.78)	17.67 (6.46)
Employment status				
Employed ^∇^	85	1619.11 (1386.16)	4.23 (2.08)	17.34 (6.62)
Unemployed	25	893.48 (545.58) **	2.83 (1.68) **	11.20 (5.86) ***
Disabled	12	673.99 (477.33) *	2.80 (1.95) *	9.35 (5.70) ***
Retired or other	5	1256.27 (1210.21)	3.47 (1.88)	11.95 (7.42)
Body Mass Index (BMI)				
Underweight ^∇^	3	903.87 (664.71)	2.86 (0.87)	14.09 (6.00)
Normal weight	39	1503.43 (1058.71)	4.75 (1.87)	18.49 (6.39)
Overweight	39	1537.17 (1449.35)	3.60 (1.76)	15.38 (5.97)
Obesity class I	9	1477.74 (1204.48)	4.56 (2.93)	16.40 (10.09)
Obesity class II	9	733.94 (673.18)	3.33 (2.01)	11.19 (5.82)
Obesity class III	28	1183.23 (1334.02)	2.67 (1.94)	11.06 (6.74)
Household level				
Annual household income (USD)				
Less than 40,000 ^∇^	62	1123.45 (1132.43)	3.27 (1.94)	12.23 (6.78)
40,001–80,000	51	1569.10 (1266.46)	4.08 (2.08) *	17.48 (6.13) ***
More than 80,000	14	1760.90 (1451.47)	4.95 (1.98) **	19.40 (6.75) ***
Housing arrangement				
Own Home ^∇^	30	1845.96 (1383.33)	4.95 (1.66)	19.01 (5.28)
Rent Home	81	1242.47 (1218.18) *	3.42 (2.03) ***	14.07 (7.25) ***
Live with Family	11	1280.89 (929.01)	3.84 (2.48)	14.74 (7.50)
Other	5	844.34 (573.61)	2.36 (1.24) **	9.61 (4.38) **
Household size				
One person ^∇^	11	778.03 (602.39)	2.94 (1.61)	11.49 (4.35)
Two people	27	1131.25 (1078.47)	2.97 (1.87)	10.97 (6.39)
Three people	36	1384.44 (1231.52)	4.29 (2.33)	17.34 (8.04) *
Four people	35	1669.35 (1504.20) *	3.76 (1.97)	15.42 (5.73)
Five people	10	1462.35 (1374.96)	4.31 (1.93)	17.56 (7.43) *
>Five people	8	1542.21 (754.39)	4.61 (1.76)	18.90 (6.18) *
Food assistance participation				
WIC Program ^∇^	73	1179.71 (1013.37)	3.39 (2.25)	13.29 (7.82)
SNAP Program	44	2292.84 (1312.08)	5.46 (1.55) *	20.69 (4.13) *
Free/reduced school meals	68	1460.69 (1370.62)	3.95 (2.17)	15.08 (7.94)
Other	9	1378.77 (1055.79)	4.77 (1.83)	16.84 (9.47)
Food security status				
High/marginal food security ^∇^	48	1323.22 (1149.14)	4.19 (2.18)	17.16 (6.98)
Low food security	26	1020.38 (1201.08)	3.15 (2.09) *	12.75 (7.18) *
Very low food security	53	1590.30 (1313.80)	3.72 (1.88)	14.44 (6.77)

^∇^ Reference group. * Statistically significant association (*p*-value < 0.05) compared with the reference group. ** Statistically significant association (*p*-value < 0.01) compared with the reference group. *** Statistically significant association (*p*-value < 0.001) compared with the reference group.

**Table 2:** Factors associated with beverage, fruit and vegetable, and fiber intake.

Characteristics	Beverages, kcal	Fruits and Vegetables, Servings	Total Fiber, Grams
	β (95% CI)	β (95% CI)	β (95% CI)
Gender	-	−0.63 (−1.31, 0.05)	6.12 (4.02, 8.22) *
Age	-	-	−2.40 (−4.38, −0.42) *
Race/ethnicity	−456.74 (−891.73, −21.74) *	−0.63 (−1.31, 0.05)	−2.40 (−4.39, −0.40) *
BMI category	-	−0.61 (−1.32, 0.09)	−1.93 (−3.97, 0.11)
Marital status	-	-	-
Education level	-	0.51 (0.12, 0.91) *	1.08 (−0.09, 2.25)
Employment status	-	-	-
Annual income	255.58 (−136.83, 647.98)	-	-
Housing arrangement	−349.67 (−695.64, −3.71) *	−0.60 (−1.09, −0.11) *	−1.77 (−3.19, −0.35) *
Household size	-	0.19 (−0.07, 0.45)	0.84 (0.08, 1.61) *
Food security status	592.87 (99.43, 1086.32) *	-	-
Children in home	541.48 (66.87, 1016.08) *	-	-
Blood pressure	−379.16 (−863.57, 105.25)	-	-
Food spending	-	0.64 (−0.01, 1.28)	1.86 (−0.06, 3.77)
Food assistance			
SNAP	-	-	-
WIC	318.77 (−124.11, 761.66)	1.07 (0.37, 1.78) *	3.22 (1.16, 5.28) *
Food intentions			
Milk	-	-	-
Bread	-	-	-
Rice	422.73 (−88.34, 933.80)	-	-
Corner store shopping			
Daily	-	-	-
Weekly	-	-	-
Supermarket shopping	-	-	-

* Statistically significant (*p*-value < 0.05).

**Table 3:** Key themes (*n* = 22) and relevant examples from respondent quotations.

Theme	Example
Staple foods	*“…pantry staples like rice, beans, flour, etc.”*
Fresh foods	*“The closest corner [store] near me had no fresh produce at the height of the pandemic which forced me to have to go to the nearest supermarket which is at least a mile from my house.”*
Online food shopping	*“I have observed that not only me tend to shop sometimes online that a lot more people in my neighborhood tends to do that now and all this came about due to the COVID-19 pandemic that limited one from going to the corner stores in person.”*
Store operation changes	*“When I go out, my options are limited. Because so many places closed down or restricted business operations during the pandemic, there are fewer options for me to get what I need.”*
Store cleanliness and sanitary practices	*“Pre COVID me didn’t really care much about cleanliness and packaging of products but after COVID started, I look out for stores that are not known for taking due process in [cleanliness].”*
Concern for expiration of perishable foods	*“The food is really not good some is expired…”*
Money spent on food	*“We save the bigger grocery stores for larger trips only once or twice a month.”*
Changes in price and affordability of food	*“We try to eat healthy but the prices have went up so much. It seems as if fruits and vegetables cost more than unhealthy foods.”*
Use of food assistance programs and pantries	*“[I] saw an increase in people relying on food pantries and corner stores for quick, cheap, or free foods”*
Proximity to stores and access to transportation	*“[Corner stores] get food from other stores and sell it to us for a much higher price than at the original store [but] most of them are far to get to if you don’t drive”*
Adoption of new cooking methods	*“I began trying out new recipes and cooking more meals at home due to restaurant closures.”*
Reduced income and job loss	*“I was shopping in bulk to try to stretch my money and feed my family”*
Fear of COVID-19 infection	*“Makes me want to not shop around other people or just go to the nearest store for something fast.”*
Food deserts	*“The pandemic has created another food desert in my community. The closest market was Save A Lot and they closed and replaced it with Dollar General. So no fresh fruits or veggies…”*
Customer foot traffic in stores	*“For me personally, I’ve had to make some pretty drastic changes to my grocery routine…now I’m finding that [some] stores are too crowded with people looking for deals.”*
Relationship between store owners and their customers	*“We did not really pay much attention to the stores around our place before the pandemic and after the pandemic we started patronizing the stores around. Now we are known more by the [store owners].”*
Last resort measures to obtain food	*“People have to be more creative about how they access food.”*
Community togetherness and support for local businesses	*“When I do go grocery shopping, I try to stick with local businesses where possible…this helps keep money circulating in our communities…”*
Changes to food sourcing and shopping patterns	*“I don’t buy the things I want as much anymore just the things I need.”*
Limited food variety and quality	*“I shop for meat bread water and fresh veggies if available [but] where I live there is not that many options.”*
Increased awareness of health	*“[People] are more concerned about healthy purchases than they used to”*
No post-pandemic changes to food sourcing or consumption made	*“…the pandemic didn’t really affect my grocery shopping habits. I shop for quick meals when I am on the go and groceries when I can get to the store.”*

## Data Availability

The original contributions presented in the study are included in the article, further inquiries can be directed to the corresponding author/s.

## References

[1] National Center for Chronic Disease Prevention and Health Promotion [NCCDPHP] Chronic Diseases in America. 13 December 2022. https://www.cdc.gov/chronic-disease/data-research/facts-stats/index.html.

[2] Ansah J.P., Chiu C.-T. (2023). Projecting the chronic disease burden among the adult population in the United States using a multi-state population model. Front. Public. Health.

[3] Gropper S.S. (2023). The role of nutrition in chronic disease. Nutrients.

[4] Kaiser Family Foundation [KFF] Key Data on Health and Health Care by Race and Ethnicity. 15 March 2023. https://www.kff.org/racial-equity-and-health-policy/report/key-data-on-health-and-health-care-by-race-and-ethnicity/.

[5] Cooksey Stowers K., Jiang Q., Atoloye A.T., Lucan S., Gans K. (2020). Racial differences in perceived food swamp and food desert exposure and disparities in self-reported dietary habits. Int. J. Environ. Res. Public Health.

[6] Park J., Kim C., Son S. (2022). Disparities in food insecurity during the COVID-19 pandemic: A two-year analysis. Cities.

[7] Thorndike A.N., Gardner C.D., Kendrick K.B., Seligman H.K., Yaroch A.L., Gomes A.V., Ivy K.N., Scarmo S., Cotwright C.J., Schwartz M.B. (2022). Strengthening US food policies and programs to promote equity in nutrition security: A policy statement from the American Heart Association. Circulation.

[8] Economos C.D., Hyatt R.R., Must A., Goldberg J.P., Kuder J., Naumova E.N., Collins J.J., Nelson M.E. (2013). Shape Up Somerville two-year results: A community-based environmental change intervention sustains weight reduction in children. Prev. Med..

[9] Ewart-Pierce E., Mejía Ruiz M.J., Gittelsohn J. (2016). “Whole-of-Community” obesity prevention: A review of challenges and opportunities in multilevel, multicomponent interventions. Curr. Obes. Rep..

[10] Gittelsohn J., Trude A.C., Poirier L., Ross A., Ruggiero C., Schwendler T., Steeves E.A. (2017). The impact of a multi-level multi-component childhood obesity prevention intervention on healthy food availability, sales, and purchasing in a low-income urban area. Int. J. Environ. Res. Public Health.

[11] Vo L., Albrecht S.S., Kershaw K.N. (2019). Multilevel interventions to prevent and reduce obesity. Curr. Opin. Endocr. Metab. Res..

[12] Gittelsohn J., Lewis E.C., Martin N.M., Zhu S., Poirier L., Van Dongen E.J.I., Ross A., Sundermeir S.M., Labrique A.B., Reznar M.M. (2022). The Baltimore Urban Food Distribution (BUD) app: Study protocol to assess the feasibility of a food systems intervention. Int. J. Environ. Res. Public Health.

[13] Ross A., Krishnan N., Panchal J., Brooks J.K., Lloyd E., Lee T.-H.J., Gittelsohn J. (2019). Formative research for an innovative smartphone application to improve distribution of healthy foods to corner stores in Baltimore City. Ecol. Food Nutr..

[14] Lewis E.C., Zhu S., Oladimeji A.T., Igusa T., Martin N.M., Poirier L., Trujillo A.J., Reznar M.M., Gittelsohn J. (2024). User experience and interface design of an innovative digital application to facilitate group purchasing and delivery of healthy foods between urban retailers and local suppliers in Baltimore City, Maryland. mHealth.

[15] Maryland Food Bank (2024). Root Causes of Hunger Research: An MFB Strategy Group Research Report. https://mdfoodbank.org/wp-content/uploads/2024/02/Maryland-Food-Bank-Root-Causes-of-Hunger-Research-Report.pdf.

[16] Misiaszek C., Buzogany S., Freishtat H. (2018). Baltimore City’s Food Environment Report: 2018 Report.

[17] Lewis E.C., Pei X., Stephenson J., Poirier L., Gittelsohn J. (2023). P22-034-23 Adapting a healthy food access adult consumer impact questionnaire for online use to better reach an under-resourced urban community. Curr. Dev. Nutr..

[18] Gittelsohn J., Anderson Steeves E., Mui Y., Kharmats A.Y., Hopkins L.C., Dennis D. (2014). B’More healthy communities for kids: Design of a multi-level intervention for obesity prevention for low-income African American children. BMC Public Health.

[19] Centers for Disease Control and Prevention Defining Adult Overweight & Obesity. Updated 3 June 2022. https://www.cdc.gov/obesity/php/about/index.html.

[20] Blumberg S.J., Bialostosky K., Hamilton W.L., Briefel R.R. (1999). The effectiveness of a short form of the Household Food Security Scale. Am. J. Public Health.

[21] Hedrick V.E., Savla J., Comber D.L., Flack K.D., Estabrooks P.A., Nsiah-Kumi P.A., Ortmeier S., Davy B.M. (2012). Development of a brief questionnaire to assess habitual beverage intake (BEVQ-15): Sugar-sweetened beverages and total beverage energy intake. J. Acad. Nutr. Diet..

[22] Fausnacht A.G., Myers E.A., Hess E.L., Davy B.M., Hedrick V.E. (2020). Update of the BEVQ-15, a beverage intake questionnaire for habitual beverage intake for adults: Determining comparative validity and reproducibility. J. Hum. Nutr. Diet..

[23] Block G., Sternfeld B., Block C.H., Block T.J., Norris J., Hopkins D., Quesenberry C.P., Husson G., Clancy H.A. (2008). Development of Alive! (A Lifestyle Intervention Via Email), and its effect on health-related quality of life, presenteeism, and other behavioral outcomes: Randomized controlled trial. J. Med. Internet Res..

[24] Lalonde I., Graham M., Slovinec-D’Angelo M., Beaton L., Brown J., Block T. (2008). Validation of the block fat/sugar/fruit/vegetable screener in a cardiac rehabilitation setting. J. Cardiopulm. Rehabil. Prev..

[25] Griffin M., Martino R.J., LoSchiavo C., Comer-Carruthers C., Krause K.D., Stults C.B., Halkitis P.N. (2022). Ensuring survey research data integrity in the era of internet bots. Qual. Quant..

[26] Creswell J.W., Plano Clark V.L., Gutmann M.L., Hanson W.E., Tashakkori A., Teddlie C. (2003). Advances in mixed methods research designs. Handbook of Mixed Methods in Social and Behavioral Research.

[27] Morse J.M., Morse J.M. (1994). Qualitative research: Fact or fantasy?. Critical Issues in Qualitative Research Methods.

[28] Castro F.G., Kellison J.G., Boyd S.J., Kopak A. (2010). A Methodology for conducting integrative mixed methods research and data analyses. J. Mix. Methods Res..

[29] Microsoft Corporation (2024). Microsoft Excel for Mac (Version 16.81). https://office.microsoft.com/excel.

[30] StataCorp (2023). Stata Statistical Software: Release 18.

[31] ATLAS.ti (2024). Scientific Software Development GmbH. ATLAS.ti Web (Version 24) [Qualitative Data Analysis Software].. https://atlasti.com.

[32] Charmaz K., Brindle P., Bryant A., Clarke A., Olesen V. (2006). Coding in Grounded Theory Practice. Constructing Grounded Theory: A Practical Guide through Qualitative Analysis.

[33] U.S. Census Bureau (2023). Estimate of Median Household Income for Baltimore City, MD [MHIMD24510A052NCEN].

[34] USDA Dietary Guidelines for Americans. https://www.dietaryguidelines.gov/sites/default/files/2019-05/Using%20Food%20Guide.pdf.

[35] (2019). Harvard Health Publishing, Harvard Medical School. Should I Be Eating More Fiber?. https://www.health.harvard.edu/blog/should-i-be-eating-more-fiber-2019022115927.

[36] Martínez Steele E., Baraldi L.G., da Costa Louzada M.L., Moubarac J.-C., Mozaffarian D., Monteiro C.A. (2016). Ultra-processed foods and added sugars in the US diet: Evidence from a nationally representative cross-sectional study. BMJ Open.

[37] Wainer A., Zabel J. (2020). Homeownership and wealth accumulation for low-income households. J. Hous. Econ..

[38] Pollack C.E., Chideya S., Cubbin C., Williams B., Dekker M., Braveman P. (2007). Should health studies measure wealth? A systematic review. Am. J. Prev. Med..

[39] Braveman P., Acker J., Arkin E., Proctor D. (2018). Wealth Matters for Health Equity.

[40] Bottino C.J., Fleegler E.W., Cox J.E., Rhodes E.T. (2019). The relationship between housing instability and poor diet quality among urban families. Acad. Pediatr..

[41] Bruce M.A.P., Thorpe R.J.J., Beech B.M.D., Towns T., Odoms-Young A. (2018). Sex, race, food security, and sugar consumption change efficacy among low-income parents in an urban primary care setting. Fam. Community Health.

[42] Fernández C.R., Chen L., Cheng E.R., Charles N., Meyer D., Monk C., Baidal J.W. (2020). Food insecurity and sugar-sweetened beverage consumption among WIC-enrolled families in the first 1000 days. J. Nutr. Educ. Behav..

[43] Mei J., Fulay A.P., Wolfson J.A., Leung C.W. (2021). Food insecurity and dietary intake among college students with unlimited meal plans at a large, midwestern university. J. Acad. Nutr. Diet..

[44] Moran A.J., Subramanian S.V., Rimm E.B., Bleich S.N. (2019). Characteristics associated with household purchases of sugar-sweetened beverages in US restaurants. Obesity.

[45] Anderson C.E., Au L.E., Yepez C.E., Ritchie L.D., Tsai M.M., Whaley S.E. (2023). Increased WIC cash value benefit is associated with greater amount and diversity of redeemed fruits and vegetables among participating households. Curr. Dev. Nutr..

[46] Carlson S., Neuberger Z. (2021). WIC Works: Addressing the Nutrition and Health Needs of Low-Income Families for More Than Four Decades.

[47] USDA National and State Level Estimates of WIC Eligibility and Program Reach in 2021. Updated 22 February 2024. https://www.fns.usda.gov/research/wic/eligibility-and-program-reach-estimates-2021.

[48] USDA National and State Level Estimates of WIC Eligibility and Program Reach in 2020. Updated 3 November 2023. https://www.fns.usda.gov/research/wic/eligibility-and-program-reach-estimates-2020.

[49] Olfert M.D., Barr M.L., Charlier C.M., Famodu O.A., Zhou W., Mathews A.E., Byrd-Bredbenner C., Colby S.E. (2018). Self-reported vs. measured height, weight, and BMI in young adults. Int. J. Environ. Res. Public Health.

[50] Zhu S., Mitsinikos C., Poirier L., Igusa T., Gittelsohn J. (2021). Development of a system dynamics model to guide retail food store policies in Baltimore City. Nutrients.

